# Ventricular septal defect characteristics influence presence of septal anatomical isthmuses in patients with repaired tetralogy of Fallot

**DOI:** 10.1093/europace/euae283

**Published:** 2024-12-16

**Authors:** Justin Wallet, Yoshitaka Kimura, Nico A Blom, Mark G Hazekamp, Margot M Bartelings, Monique R M Jongbloed, Katja Zeppenfeld

**Affiliations:** Department of Cardiology, Heart Lung Centre, Leiden University Medical Centre, P.O. Box 9600, 2300 RC Leiden, The Netherlands; Willem Einthoven Centre of Arrhythmia Research and Management (WECAM), Leiden, The Netherlands; Centre for Congenital Heart Disease Amsterdam-Leiden (CAHAL), Leiden, The Netherlands; Department of Cardiology, Heart Lung Centre, Leiden University Medical Centre, P.O. Box 9600, 2300 RC Leiden, The Netherlands; Willem Einthoven Centre of Arrhythmia Research and Management (WECAM), Leiden, The Netherlands; Centre for Congenital Heart Disease Amsterdam-Leiden (CAHAL), Leiden, The Netherlands; Centre for Congenital Heart Disease Amsterdam-Leiden (CAHAL), Leiden, The Netherlands; Department of Paediatric Cardiology, Leiden University Medical Centre, Leiden, The Netherlands; Centre for Congenital Heart Disease Amsterdam-Leiden (CAHAL), Leiden, The Netherlands; Department of Cardiothoracic Surgery, Leiden University Medical Centre, Leiden, The Netherlands; Centre for Congenital Heart Disease Amsterdam-Leiden (CAHAL), Leiden, The Netherlands; Department of Anatomy & Embryology, Leiden University Medical Centre, Leiden, The Netherlands; Department of Cardiology, Heart Lung Centre, Leiden University Medical Centre, P.O. Box 9600, 2300 RC Leiden, The Netherlands; Centre for Congenital Heart Disease Amsterdam-Leiden (CAHAL), Leiden, The Netherlands; Department of Anatomy & Embryology, Leiden University Medical Centre, Leiden, The Netherlands; Department of Cardiology, Heart Lung Centre, Leiden University Medical Centre, P.O. Box 9600, 2300 RC Leiden, The Netherlands; Willem Einthoven Centre of Arrhythmia Research and Management (WECAM), Leiden, The Netherlands; Centre for Congenital Heart Disease Amsterdam-Leiden (CAHAL), Leiden, The Netherlands

**Keywords:** Tetralogy of Fallot, Congenital heart disease, Ventricular septal defect, Ventricular tachycardia, Morphology

## Abstract

**Aims:**

In repaired tetralogy of Fallot (rTOF), the septal anatomical isthmuses (AI), AI 3, between the ventricular septal defect (VSD) and pulmonary annulus, and AI 4, between the VSD and tricuspid annulus, are important ventricular tachycardia (VT) substrates when slow conducting. Our aim was to assess the influence of VSD characteristics, specifically the presence of muscular or fibrous tissue at its border, on the presence or absence of septal AIs, potentially related to VT.

**Methods and results:**

All consecutive rTOF patients who underwent electroanatomical mapping between January 2005 and March 2023 with an available surgical report providing VSD details (*n* = 91) were included. The majority of patients had an outlet perimembranous VSD (*n* = 76, 84%), 6 (7%) an outlet muscular VSD, and 7 (8%) a doubly committed juxta-arterial VSD. In patients with an outlet perimembranous VSD, AI 3 was present in almost all (97%), whereas no AI 4 was observed. In patients with an outlet muscular VSD, AI 3 and AI 4 were present in 83% and 33%, respectively. In all patients with a doubly committed VSD, where the outlet septum is hypoplastic/fibrous, AI 3 was absent. Among patients with a doubly committed VSD with a muscular postero-inferior rim, 50% had AI 4, whereas none of those with a fibrous postero-inferior rim had AI 4.

**Conclusion:**

Ventricular septal defect characteristics aid in determining the presence of septal AIs potentially related to VT. In patients with doubly committed VSDs, septal VT substrates are unlikely. Detailed knowledge of anatomical VSD characteristics is desirable for understanding VT in rTOF.

Ventricular tachycardias (VT) occur in patients with repaired tetralogy of Fallot (rTOF) typically from mid-adulthood to older ages.^1,2^ Electroanatomical mapping (EAM) studies have identified anatomical isthmuses (AI), defined as corridors of excitable myocardium bordered by unexcitable tissue, as dominant substrate of VT if slow conducting. As concerns have been raised that slow conducting AI can become inaccessible after pulmonary valve replacement, these substrates are increasingly identified and targeted by ablation before re-valving.^3–8^ Slow conducting AI 3, between the ventricular septal defect (VSD) patch and pulmonary annulus, remains the most prevalent substrate for spontaneous and inducible VT in rTOF patients.^4–6,9,10^ AI 4, between the VSD patch and tricuspid annulus, is reported in 0–5% of rTOF patients and is in close proximity to the conduction system.^4–6,9,11^ In 10% of patients, EAM demonstrates that the area of an expected AI 3 consists of unexcitable tissue, excluding its contribution to arrhythmogenicity.^9^ Tetralogy of Fallot is characterized by antero-cephalad deviation of the outlet septum leading to subaortic malalignment VSD, of which anatomical variations have been described.^11–13^ The aim of this study was to determine if VSD characteristics, as described by the surgeons during initial repair, are associated with the presence of septal AIs.

Consecutive adults and children with rTOF who underwent EAM at the Leiden University Medical Centre for treatment of VT, prior to re-valving, or for risk stratification, between January 2005 and March 2023, with an available surgical report of initial repair describing VSD characteristics, were included. Patients provided informed consent before the procedure. The study complies with the Declaration of Helsinki and was approved by the Medical Ethics Committee Leiden The Hague Delft (GP21.137).

The presence of an AI was defined as electrical excitable tissue bordered by unexcitable boundaries determined by EAM.^4,9^ A slow conducting AI was defined based on a conduction velocity of <0.5 m/s during baseline rhythm or pacing.^4^ Tetralogy of Fallot with double outlet right ventricle was defined as overriding of the aorta > 50% (*n* = 12, none with a bilateral conus).^14^ Surgical reports of initial repair were scrutinized for VSD characteristics, and the most suited VSD type was selected from the classification document of the International Society for Nomenclature of Paediatric and Congenital Heart Disease.^12^ Outlet perimembranous defects are characterized by a subaortic location and a *fibrous* postero-inferior border in continuity with the fibrous tricuspid annulus. The infundibular septum, located cranially, between the defect and the pulmonary annulus (=AI 3), is muscular. In an outlet muscular VSD, the defect is located between the limbs of the septal band and both the outlet septum (=AI 3) and the postero-inferior rim are muscular (=AI 4).^12,13^ Doubly committed juxta-arterial defects (henceforth ‘doubly committed VSD’) have a fibrous outlet septum (also described as hypoplastic or ‘absent’). The cranial border of this VSD is the area of fibrous continuity between the pulmonary and aortic annuli. The postero-inferior rim of this defect can be in fibrous continuity to the tricuspid annulus, or muscular (=AI 4) (see *Figure 1*).

**Figure 1:**
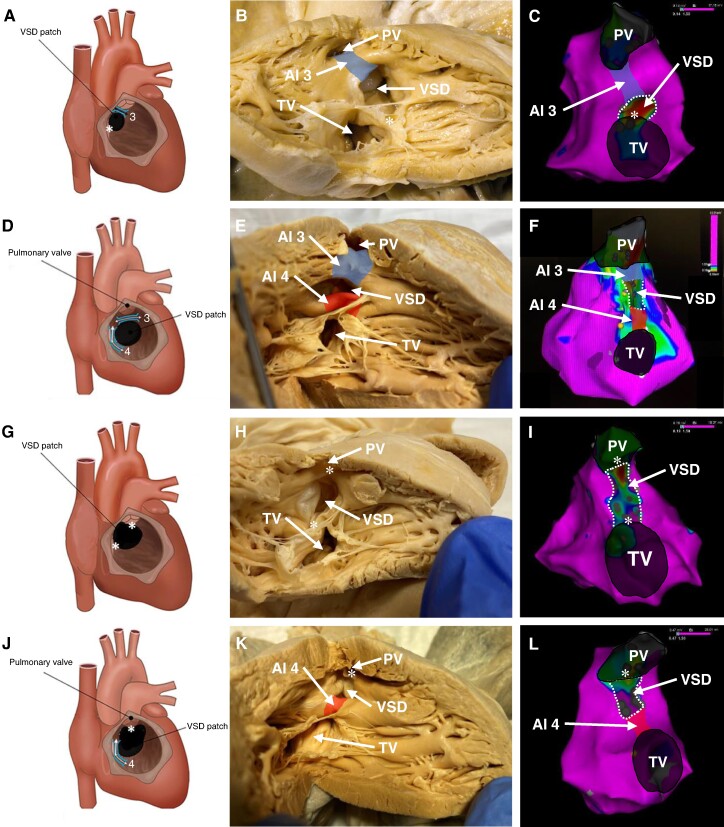
(*A–C*) Tetralogy of Fallot patient with an outlet perimembranous VSD. (*A*) Schematic, the right ventricle is incised and the right ventricular septum, including the VSD and septal isthmus area, is exposed. (*B*) Anatomical specimen. (*C*) Electroanatomical voltage map, colour-coded according to bar. (*D–F*) Patient with an outlet muscular VSD. AI 3 and AI 4 are present. Both isthmuses in (*F*) were slow conducting. (*G–I*) Patient with a doubly committed juxta-arterial VSD with fibrous postero-inferior rim (indicated by asterisks). Note the absence of all septal AIs. (*J–L*) Patient with a doubly committed juxta-arterial VSD with muscular postero-inferior rim. Note the absence of AI 3 and presence of AI 4. The anatomical specimens, all from the Leiden Collection of malformed hearts, and the electroanatomical voltage maps depict different patients with the same VSD characteristics. In the schematics, the AIs are indicated by the white arrows flanked by the blue brackets. In the anatomical specimens and the electroanatomical map, AI 3 is indicated by the blue area and AI 4 by the red area. *Fibrous continuity. AI, anatomical isthmus; PV, pulmonary valve; VSD, ventricular septal defect; TV, tricuspid valve. Schematics (*A*), (*D*), (*G*), and (*J*) adapted from Zeppenfeld K, Wijnmaalen AP. Clinical aspects and ablation of ventricular arrhythmias in tetralogy of Fallot. Card Electrophysiol Clin. Jun 2017;9(2):285–294; by permission of Elsevier. This content (*A*, *D*, *G*, and *J*) is not covered by the terms of the Creative Commons license of this publication. For permission to reuse, please contact the rights holder.

In 132 of the 154 patients, a surgical report was available, which provided VSD details in 91. Thirty-five per cent were female, the median age at repair was 2.0 years (interquartile range 0.7–5.9), and the median age at EAM was 35 years (interquartile range 16–45). The majority of patients had an outlet perimembranous VSD (*n* = 76, 84%). Among those, at EAM, AI 3 was detected in 74 (97%). None had AI 4. Six patients had an outlet muscular VSD. At EAM, in 5/6, AI 3 could be identified and AI 4 was present in 2/6. Seven patients had a doubly committed VSD, of whom three had a fibrous, two a muscular, and two an unknown postero-inferior rim. In all patients, AI 3 was absent at EAM. Of note, among all patients with an absent AI 3 (*n* = 10), 70% had a doubly committed VSD. AI 4 was absent in the two patients with a fibrous postero-inferior margin. AI 4 was also absent in one with a muscular and one with unknown properties of the postero-inferior rim. Presence of slow conducting properties of the AI can be appreciated from *Table 1*.

**Table 1:** Overview of ventricular septal defect characteristics and the presence of septal anatomical isthmuses

Classification	Subclassification	Postero-inferior rim	*n*	AI 3	AI 4	*n*	AI 3/4 in %
Outlet VSD with anteriorly malaligned outlet septum	Outlet perimembranous	–	76	NCAI 3	–	46	AI 3 in 97% AI 4 in 0%
				SCAI 3	–	28	
				Absent	–	2	
	Outlet muscular	–	6	NCAI 3	–	2	AI 3 in 83% AI 4 in 33%
				SCAI 3	–	1	
				SCAI 3	SCAI 4	1	
				SCAI 3	NCAI 4	1	
				Absent	–	1	
	Doubly committed juxta-arterial	Fibrous postero-inferior rim	3	Absent	–	3^a^	AI 3 in 0% AI 4 in 0%
		Muscular postero-inferior rim	2	Absent	–	1	AI 3 in 0% AI 4 in 50%
				Absent	NCAI 4	1	
		Unknown postero-inferior rim	2	Absent	–	1	AI 3 in 0% AI 4 in 50%
				Absent	SCAI 4	1	
TOF with inlet VSD	–	–	1	NCAI 3	–	1	N/A
TOF with AVSD	–	–	1	SCAI 3	–	1	N/A

SCAI/NCAI, slow/normal conducting anatomical isthmus (AI); (A)VSD, (atrio)ventricular septal defect.

^a^One patient with probably blocked AI 3.

This is the first study that evaluates the association between VSD morphology, as available from surgical reports at the time of repair, and the presence of septal AIs and their conduction properties as potential VT substrate, in rTOF at EAM later in life. Surgical description of fibrous tissue in continuity with valve annuli at the VSD border excluded the presence of an AI. However, despite the description of muscular tissue at the postero-inferior rim during repair, EAM did not show the anticipated presence of AI 4 in 5 of the 8 patients. An expected AI 3 was not found in 3 of the 82 patients (*n* = 2 outlet perimembranous and *n* = 1 outlet muscular VSD).

Of note, EAM was performed many years after repair and AI myocardium may have remodelled over time due to aging and as a consequence of interventions such as extensive infundibular resection, suture placement, and stent insertion. Progressive fibrosis may lead to slow conducting AI or complete conduction block, in particular if AIs have reduced wall thickness.^15^ Indeed, histological studies have demonstrated post-surgical fibrosis despite an apparently intact muscular band at initial repair.^11^

As no septal AIs are expected in patients with doubly committed VSDs with a fibrous postero-inferior rim, one could advocate to omit EAM before re-valving.^3,4^ In those with an outlet muscular VSD, where AI 3 and AI 4 can be simultaneously present, slow conduction across one can be difficult to detect and may be missed without the information from the surgical report. Our results emphasize the importance of accurate description of VSD characteristics in the surgical report, which should also include the widths and wall thickness of AI to better understand remodelling over time.

## Conclusion

Anatomical variations in VSD morphology, as known from the initial repair, influence the presence of AI 3 and AI 4 in patients with rTOF. Reporting VSD details at initial repair and knowledge of the variations can refine patient selection for invasive mapping prior to re-valving, facilitates mapping, and is important for the understanding of potential VT substrates in rTOF.

## Data Availability

The data underlying this article will be shared on reasonable request to the corresponding author.
